# Early effective intervention can significantly reduce all-cause mortality in prediabetic patients: a systematic review and meta-analysis based on high-quality clinical studies

**DOI:** 10.3389/fendo.2024.1294819

**Published:** 2024-03-01

**Authors:** Xuedong An, Yuehong Zhang, Wenjie Sun, Xiaomin Kang, Hangyu Ji, Yuting Sun, Linlin Jiang, Xuefei Zhao, Qing Gao, Fengmei Lian, Xiaolin Tong

**Affiliations:** ^1^ Guang’anmen Hospital, China Academy of Chinese Medical Sciences, Beijing, China; ^2^ Fangshan Hospital of Beijing University of Chinese Medicine, Beijing, China

**Keywords:** prediabetes, early intervention, cardiovascular disease, meta-analysis, lifestyle

## Abstract

**Background:**

Reducing the occurrence of diabetes is considered a primary criterion for evaluating the effectiveness of interventions for prediabetes. There is existing evidence that early lifestyle-based interventions can significantly decrease the incidence of diabetes. However, whether effective interventions can reduce long-term outcomes in patients, including all-cause mortality, cardiovascular risks, and the occurrence of microvascular complications, which are the most concerning issues for both patients and clinicians, remains a subject of inconsistent research findings. And there is no direct evidence to answer whether effective intervention has long-term benefits for prediabetic patients. Therefore, we conducted a systematic review and meta-analysis to assess the relationship between early effective intervention and macrovascular and microvascular complications in prediabetic patients.

**Methods:**

PubMed, Embase, and Cochrane Central Register of Controlled Trials were searched for the randomized controlled trials of lifestyle or/and drugs intervention in prediabetes from inception to 2023.9.15. Two investigators independently reviewed the included studies and extracted relevant data. Random or fixed effects model meta-analysis to derive overall relative risk (RR) with 95% CI for all-cause mortality, cardiovascular events, and microvascular complications.

**Results:**

As of September 15, 2023, a total of 7 effective intervention studies were included, comprising 26 articles out of 25,671 articles. These studies involved 26,389 patients with a total follow-up duration of 178,038.6 person-years. The results indicate that effective intervention can significantly reduce all-cause mortality in prediabetic patients without a history of cardiovascular disease by 17% (RR 0.83, 95% CI 0.70-0.98). Additionally, effective intervention reduced the incidence of retinopathy by 38% (RR 0.62, 95% CI 0.70-0.98). Furthermore, the study results suggest that women and younger individuals have lower all-cause mortality and cardiovascular mortality. Subsequently, we conducted an in-depth analysis of patients without a history of cardiovascular disease. The results revealed that prediabetic patients with a 10-year cardiovascular risk >10% experienced more significant benefits in terms of all-cause mortality (*P*=0.01). When comparing the results of all-cause mortality and cardiovascular mortality from the Da Qing Diabetes Prevention Outcome Study longitudinally, it was evident that the duration of follow-up is a key factor influencing long-term benefits. In other words, the beneficial effects become more pronounced as the intervention duration reaches a certain threshold.

**Conclusion:**

Early effective intervention, which significantly reduces the incidence of diabetes, can effectively lower all-cause mortality in prediabetic patients without a history of cardiovascular disease (especially those with a 10-year cardiovascular risk >10%), with women and younger individuals benefiting more significantly. Additionally, the duration of follow-up is a key factor influencing outcomes. The conclusions of this study can provide evidence-based guidance for the clinical treatment of prediabetic patients to prevent cardiovascular and microvascular complications.

**Systematic review registration:**

https://www.crd.york.ac.uk/prospero, identifier CRD42020160985.

## Introduction

Prediabetes is defined as the intermediate metabolic state between normal blood glucose levels and diabetes, including impaired glucose tolerance (IGT) and impaired fasting glucose (IFG). According to the latest estimates of the International Diabetes Federation (IDF) (2017), 352 million adults (7.3%) can be classified as prediabetes (1). Prediabetes is the risk factor for type 2 diabetes and its microvascular complications (2–4). Multiple large sample meta-analyses have shown that prediabetes is also risk factor for macroangiopathy (5, 6) and is associated with increased risk of liver cancer, endometrial cancer, and gastric/colorectal cancer (7).

Therefore, timely identification of prediabetic individuals and effective management are key to preventing the onset of diabetes. American Diabetes Association (ADA) suggests that lifestyle intervention is the basic management for prediabetes. The recommended drug intervention may consider IGT and IFG, age <60 years, body mass index (BMI) >=35kg/m^2^, family history of first-degree related diabetes, high concentration individuals with triglycerides proceed (8). Studies have shown that early intervention can significantly reduce the incidence of diabetes in patients with prediabetes (9, 10). Such as insulin-sensitizing agents and treatment that reduces postprandial hyperglycemia can reduce the risk of progression to T2DM in high-risk prediabetes subjects (11), and lifestyle intervention is the cornerstone for preventing progression to diabetes (12).

However, for prediabetes, just like diabetes, the primary focus of early intervention should be on preventing the occurrence of macrovascular and microvascular complications. So, can interventions that effectively reduce the incidence of diabetes in prediabetic patients also effectively reduce the occurrence of microvascular and macrovascular complications? Based on a comprehensive review of current evidence, the impact of effective intervention on long-term outcome measures, including all-cause mortality and cardiovascular events, remains inconsistent. For example, the Da Qing Diabetes Prevention Outcome Study (DQ) demonstrated that lifestyle intervention could effectively reduce the incidence of diabetes and suggest a reduction in all-cause mortality (13–17). The STOP-Non-insulin dependent diabetes mellitus trial (STOP), which combined lifestyle and acarbose, showed a significant reduction in cardiovascular events (18–21). However, the Acarbose Cardiovascular Evaluation Study (ACE), which applied acarbose in combination with lifestyle intervention, was effective in reducing diabetes incidence but had no effect on cardiovascular events (22, 23). Therefore, the current evidence does not provide clear guidance for clinicians regarding the long-term benefits of prediabetic patients. Hence, we aim to assess the impact of effective intervention on the long-term outcomes of prediabetic patients through a systematic review and meta-analysis.

## Methods

This study was implemented and reported in accordance with the guidelines for systematic review and meta-analysis of the preferred reporting project (PRISMA) (24). This analysis does not involve personal information, ethical approval or patient consent is exempted, and has been prospectively registered in PROSPERO: CRD42020160985.

### Data sources and searches

Pubmed, Embase and Cochrane Central Register of Controlled Trials for randomized controlled studies of prediabetes interventions were searched from inception to 2023.9.15. At the same time, references and related systematic reviews are included in the literature to help complete the search. A manual search of the references of included trials supplemented the electronic search.

### Study selection

Studies were included if they met the following PICO(S) (participants, intervention, comparators, outcomes (study designs)) criteria:

1. Participants: The participants were prediabetes (IGT and/or IFG) patients, no gender or racial restrictions, including with or without a history of cardiovascular disease.
2. Intervention: Interventions included lifestyle (including dietary and exercise recommendations) or drugs (including acarbose, metformin)。
3. Comparator: The intervention measures in the control group are either a blank control, a placebo, or lifestyle guidance.
4. Outcome: main outcome is all-cause mortality, secondary outcomes include composite cardiovascular outcomes (including cardiovascular mortality, non-fatal myocardial infarction, non-fatal stroke, heart failure hospitalization, arterial revascularization or hospitalization of unstable angina), core cardiovascular outcomes (including cardiovascular mortality, non-fatal myocardial infarction, non-fatal stroke), cardiovascular mortality, microvascular complications (retinopathy, renal disease, peripheral neuropathy).
5. Study designs: The study type was randomized controlled trial (blind or not); JADAD score between 5 and 7 points.

Studies were excluded for: duplicate publications, only the abstract or lack of data and cannot obtain full-text articles, unable to extract data for research, and non-English literature. Studies included less than 100 participants.

### Definitions of different indicators/standards

Effective Intervention: In the RCTs we included in the analysis, the intervention that can significantly reduce the occurrence of diabetes is referred to as an effective intervention, and when compared to the control group, it achieves statistical significance (*P*<0.05).

Cardiovascular History: Patients without a history of cardiovascular disease, meaning individuals included in the study who do not have obvious coronary heart disease. Studies specifically including patients with a history of coronary heart disease are classified as patients with cardiovascular history.

Age Classification: The “Report on the Nutrition and Chronic Diseases Status of Chinese Residents (2020)” indicates that being over 50 is a significant risk factor for the progression from prediabetes to diabetes. Additionally, individuals over the age of 50 tend to exhibit more abnormalities in glucose and lipid metabolism, along with a higher incidence of coronary heart disease. So, we using 50 years as the cutoff, individuals aged 50 or older are categorized as older patients, while others are considered general patients, including those younger than 50 and those older than 50.

Weight Classification: According to the World Health Organization (WHO) classification criteria for obesity, a BMI greater than 25 is considered obese. Therefore, based on a body mass index (BMI) of 25 as the threshold, individuals with a BMI greater than or equal to 25 are classified as overweight patients, while others are considered general patients, including those with a BMI less than 25 and those with a BMI greater than 25.

10-Year Cardiovascular Risk: The 10-year cardiovascular risk of the composite cardiovascular outcome was calculated by multiplying the annualized rate of cardiovascular outcome in the control group by 10 years. High cardiovascular risk is defined as the 10-year cardiovascular risk > = 10%, and low cardiovascular risk is defined as 10-year cardiovascular risk <10%.

### Data extraction and quality assessment

Screening out duplicate articles with EndNote X9, two authors (XA and YZ) extracted and checked the data with the Office form separately, included the basic characteristics of the included study (including the country where the study was carried out, the number of patients included, the basic characteristics of the included patients, study design methods, interventions, intervention time, follow-up time, main outcomes, JADAD score) and the basic characteristics of the included patients (age, gender, race, body mass index (BMI), smoking history, hypertension, dyslipidemia), if there is any objection, it would be resolved through consultation. If the result data is reported at multiple follow-up points, the data from the longest follow-up would be selected.

I^2^ and Cochran’s Q test were used to evaluate the heterogeneity of treatment effects between trials. *P <*0.05 and I^2^> 50% of the Cochran’s Q test indicated significant heterogeneity. For studies with low heterogeneity, fixed-effect model is used. Studies with greater heterogeneity, the source of heterogeneity is first searched, and a random-effects model is used for analysis. For studies with no source of heterogeneity, only descriptive analysis.

### Grading of the evidence

The quality of the studies was evaluated by JADAD 7 points (25), including the generation of random sequence, randomization hiding, blind method, withdrawal and exit. 1 to 3 is divided into low-quality study, 4 to 7 is divided into high-quality study. The two authors independently assessed the risk of publication bias in each study, and disputes were resolved through negotiation.

### Data analysis

The results were analyzed with RevMan 5.2 provided by Cochrane Collaboration (26) and Prism 9.5.1. The secondary classification results were analyzed by relative risk (RR) and 95% confidence interval (CI). According to the characteristics of the patients, including the history of cardiovascular disease, age, obesity, gender, 10-year cardiovascular risk factors, outcomes were evaluated by subgroup analysis. According to the Cochrane manual, funnel plots were used to assess potential publication bias (27). Heterogeneity was evaluated using I^2^ statistics. I^2^ <25% represents low heterogeneity, 25-50% represents medium heterogeneity, and> 50% represents high heterogeneity, considering the heterogeneity difference *P* value, if I^2^ <50% and *P*> 0.05 then Fixed effect model was used, if I^2^ > 50% and *P <*0.05, random effect model was used. For studies with large heterogeneity that cannot find the source of heterogeneity, only descriptive analysis is made.

## Results

**Figure 1:**
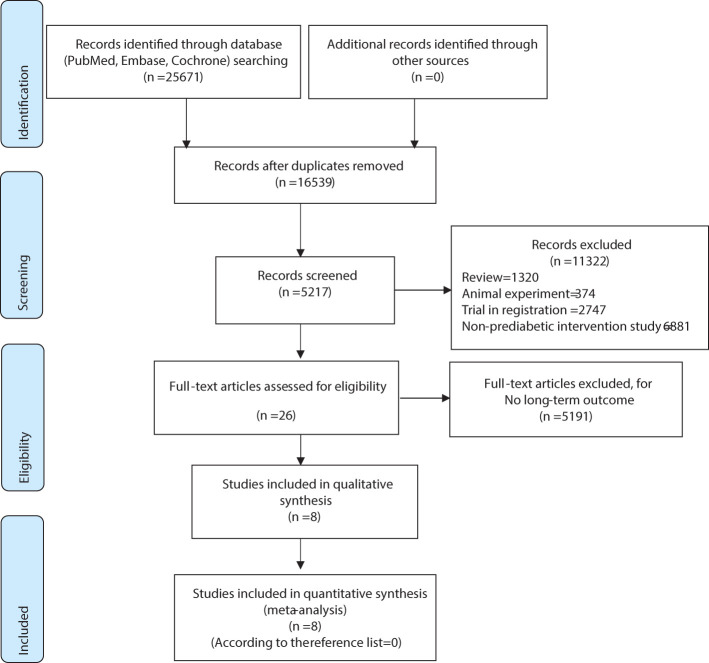
Flow Diagram.

**Figure 2:**
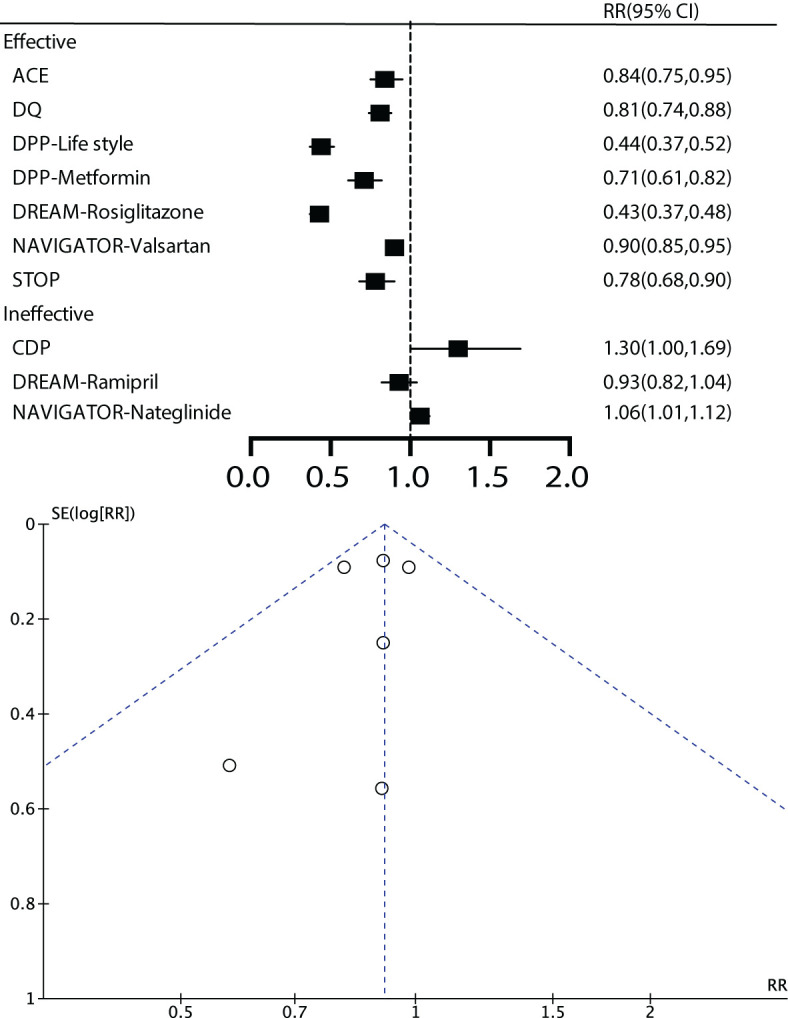
The diabetes incidence results in different studies.

As shown in **Figure 1** in the flowchart, we retrieved a total of 25,671 articles from three databases. First, we excluded duplicate articles, leaving us with 16,539 articles. Next, we reviewed titles and abstracts and excluded 11,322 unrelated articles, including 1,320 reviews, 374 animal experiments, 2,747 registered studies, and 6,881 articles without interventions. After a full-text review, we excluded 5,191 articles that lacked long-term outcome measures, leaving us with 26. No additional articles were added from the reference lists. In the end, a total of 8 studies were included for analysis, and two of these studies utilized a 2x2 factorial design. Simultaneously, we further analyzed seven studies based on the effectiveness of the intervention measures employed in the research (**Figures 1**, **2**).

### Basic characteristics of the including studies and quality evaluation

We conducted a statistical analysis of the 8 studies included, and the results indicated that, by analyzing the diabetes outcomes of prediabetic patients through early effective intervention, we initially included 7 studies. These studies encompassed, The Acarbose Cardiovascular Evaluation (ACE) (22, 23), the Da Qing Diabetes Prevention Outcome Study (DQ) (13–17), the Diabetes Prevention Program Outcomes Study (DPP) (28–30), the Diabetes Reduction Assessment with ramipril and rosiglitazone Medication (DREAM- Rosiglitazone) trial (31–34), Nateglinide and Valsartan in Impaired Glucose Tolerance Outcomes Research (NAVIGATOR- Valsartan) (35–38), the STOP-Non-insulin dependent diabetes mellitus trial (STOP) (18–21), The Finnish Diabetes Prevention Study (FDPS) (39–42) (**Table 1**). 

**Table 1:** Basic characteristics of the including studies.

Studies	Country(cite)	Total patients(T/C)	Study subject	Study design	Intervention	Intervention duration (years)	Follow-up period (years)	Diagnostic criteria	Primary outcomes	10-year Cardiovascular risk (%)
the STOP-Non-insulin dependent diabetes mellitus (NIDDM) trial (STOP) (18)	Canada, Germany, Austria, Norway, Denmark, Sweden, Finland, Israel, Spain (23)	1418(682/686)	IGT; Age 40–70 years; BMI 25–40kg/m2; FBG 5.6-7.8mmol/L	Multinational, double-blind, placebo-controlled, randomized study	T:100 mg acarbose three times/day; C:Placebo All patients received weight loss guidance or weight maintenance diets and were encouraged to engage in regular exercise.	3.3	3.3 ± 1.15	WHO1985	Cardiovascular events, hypertension	13.93
The Da Qing Diabetes Prevention Outcome Study (DQ) (13, 14)	China (33)	576(438/138)	IGT; Age>=25 years	Randomized controlled trial	Diet group counseled on healthy diet and losing weight if overweight/obese. Exercise group individually counseled on increasing physical activity. Combined group received diet & exercise counseling. Control group: General information about diabetes, IGT, general instructions for diet and/or physical activities	6	30	WHO1985	Diabetes incidence, all-cause mortality, cardiovascular disease mortality, any major vascular or microvascular complications associated with diabetes	19.75
The Diabetes Prevention Program Outcomes Study (DPP) (28)	United States (27)	2776(MET:1073; LSM:1079; C: 1082)	Age>=25years; FPG:5.3–6.9 mmol/l; 2hPG: 7.8–11.0 mmol/l; BMI>=24 kg/m2(22 in Asians);	Randomized controlled trial	MET: 850 mg twice daily; LS: individual counselling sessions on diet, exercise, and behavior modification over 24 weeks.Goals were 7% weight loss and ≥150 min physical activity per week; C:placebo tablet twice daily.	2.8	15	ADA 1997	Incidence of diabetes, cardiovascular events, blood sugar, kidney function	1.36
the Diabetes Reduction Assessment with ramipril and rosiglitazone Medication (DREAM) trial (31)	191	5269 (Rosiglitazone:2635/2634; Ramipril:2623/2646)	IGT or/and IFG, no previous diabetes or cardiovascular disease; Age>=30 years	International, multi-center, randomized and double-blind controlled trials, 2 * 2 factorial design	T: rosiglitazone 8 mg daily; C: placebo.	1	3	WHO 1999, ADA 2003	Compound cardio-renal outcome; compound renal outcome; compound cardiovascular outcome	8.06
The Finnish Diabetes Prevention Study (FDPS) (39)	Finland (5)	522 (265/257)	IGT; 40–64 years old; BMI>25 kg/m2	Randomized controlled trial	T: receive counseling from nutritionist to achieve ≥ 5% weight loss, moderate intensity physical activity ≥ 30 min/day, and fat < 30% of total calories. C: lifestyle and diabetes information in one session.	3.2	10.6	WHO 1985	development of diabetes; Cardiovascular mortality and morbidity	20.54
NAVIGATOR	40 countries	9306(Valsartan:4631/4675;Nateglinide:4645/4661)	Individuals with impaired glucose tolerance, fasting blood glucose concentration of at least 95 mg/dL (5.3 mmol/L) but less than 126 mg/dL (7.0 mmol/L), and one or more cardiovascular risk factors (for participants aged 55 or older) or known cardiovascular disease (for participants aged 50 or older).	International, multi-center, randomized and double-blind controlled trials, 2 * 2 factorial design	The intervention aims to help patients achieve and maintain a 5% reduction in weight, reduce saturated fat and total dietary fat intake, and increase physical activity to 150 minutes per week. The dose of Valsartan is 80mg once daily, increased to 160mg once daily after 2 weeks. Nateglinide is initially dosed at 30mg, increased to 60mg after 2 weeks.”	6.5	6.5	WHO 1999	diabetes and cardiovascular disease	22.80
ACE	China	3272, 3250	Age 50 or older, with coronary heart disease, and prediabetes diagnosis.	Randomized, double-blind, placebo-controlled, event-driven, phase 4 superiority trial.	Acarbose, 50mg, tid; placebo	3	5	Impaired glucose tolerance diagnosed on a single 75 g anhydrous glucose OGTT, defined as a 2-hour plasma glucose (2HPG) value ≥7.8 but b11.1 mmol/L and a FPG b7.0 mmol/L.	Diabetes incidence, cardiovascular events, mortality.	29.48

T/C, Treatment group/control group; IGT, impaired glucose tolerance; IFG, impaired fasting glucose; BMI, body mass index; FBG, Fasting blood glucose; WHO, World Health Organization; ADA, American Diabetes Association; MET, Metformin; LS, Lifestyle mearnment; NR, Not reported.

Included in the studies, the STOP study, DREAM study, NAVIGATOR study, and ACE study were randomized double-blind placebo-controlled studies, while the DQ study, DPP study, FDPS study were randomized controlled studies. Regarding the intervention measures in different studies, the DQ study and FDPS study solely involved lifestyle interventions. Studies involving drug interventions were all based on lifestyle interventions, including the STOP study (acarbose, 100mg, three times/day), DPP study (metformin, 850mg, twice/day), DREAM study (rosiglitazone 8 mg daily), NAVIGATOR study (dose of valsartan started at 80mg daily and increased to 160mg daily after 2 weeks), and ACE study (acarbose, 50mg, three times/day). We also summarized the baseline characteristics of the included studies, including age, gender ratio, ethnicity, BMI, smoking history, history of hypertension, history of lipid abnormalities, and history of cardiovascular disease (**Table 2**).

**Table 2:** Basic characteristics of the including patients.

Studies	Total(n)	Age (years)	Male (%)	Race	BMI (Kg/m2)	Current smoking (%)	Past smoking (%)	Hypertension (%)	Dyslipidemia (%)	History of cardiovascular disease (%)
STOP	1368	54.5 ± 7.9	673(49.20)	NR	30.9 ± 4.2	13.01	NR	51.32	57.68	NR
DQ	568	T:44.7 ± 9·3; C:46.6 ± 9·3	309(54.40)	NR	T:25.7 ± 3.8; C:26.2 ± 3.8	41.20	NR	NR	NR	NR
DPP	3234	50.6 ± 10.0	1043(32.3)	Caucasian: 54.7%; African-American: 19.9%; Hispanic: 15.7%; American Indian: 5.3%; Asian-American: 4.4%	34.0 ± 6.7	7.0	34.4	26.93	35.22	Myocardial infarction:1.0; Stroke 1.1
FDPS	505	T:55.4± 7.3; C:55.0± 6.9	T:34.2; C:31.5	NR	T:31.4 ± 4.6; C:31.2 ± 4.5	T:7.0; C:7.3	NR	NR	NR	T:8.2; C:8.1
DREAM- Rosiglitazone	5269	54.7 ± 10.9	40.79	NR	30.5 ± 5.1	44.60		43.48	35.32	2.51
NAVIGATOR valsartan trial	9306	T: 63.7 ± 6.8; C: 63.8 ± 6.9	4595;49	White (3854 (83.0), 3880 (83.2) )Black (120(2.6), 116(2.5));Asian (310 (6.7), 303 (6.5) );Other (361(7.8), 362(7.8))	30.5 ± 5.4	11.02		77.58%		24.36%
ACE	6522	64.3 ± 8.1	4760;73		25.4 ± 3.1	13%	47%			Myocardial infarction 42%, unstable angina 42%, stable angina 22%.

NR, Not reported; T, Treatment group; C, control group.

For the studies we included, the JADAD scores were 7 for the STOP, DREAM, NAVIGATOR, and ACE studies, and 5 for the DQ, DPP, and FDPS studies, all indicating high-quality research (**Table 3**).

**Table 3:** Publication bias (JADAD scores) of the including studies.

Studies	Generation of Random Sequences Appropriate (2 points) Uncertain (1 point) Inappropriate (0 points)	Randomization Concealment Appropriate (2 points) Uncertain (1 point) Inappropriate (0 points) Not Used (0 points)	Blinding Appropriate (2 points) Uncertain (1 point) Inappropriate (0 points)	Withdrawal and Dropout Describes the number and reasons for withdrawal or dropout (1 point) Does not describe the number or reasons for withdrawal or dropout (0 points)	Jadad score
STOP	2	2	2	1	7
DQ	2	2	0	1	5
DPP	2	2	0	1	5
DREAM	2	2	2	1	7
FDPS	2	2	0	1	5
NAVIGATOR	2	2	2	1	7
ACE	2	2	2	1	7

### Main outcome

**Figure 3:**
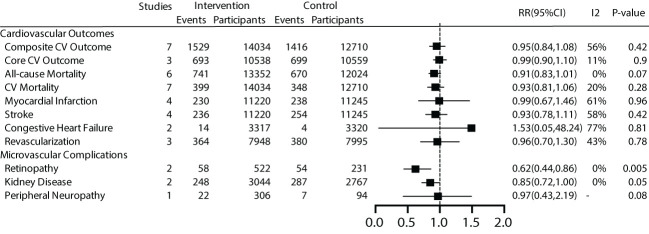
Main and secondary outcomes.

For the main outcome, namely all-cause mortality, a total of 6 studies were included, comprising 25,867 patients with a total follow-up duration of 172,505.4 person-years. The results indicated low heterogeneity among the various studies (I^2 ^= 0%, *P*=0.40). Using a fixed-effect model for analysis, the results showed that effective intervention could reduce all-cause mortality by 9% (RR 0.91, 95% CI 0.83-1.01), but it was not statistically significant (*P*=0.07). The funnel plot exhibited a symmetrical shape, suggesting the absence of publication bias (**Figure 3**).

**Figure 4:**
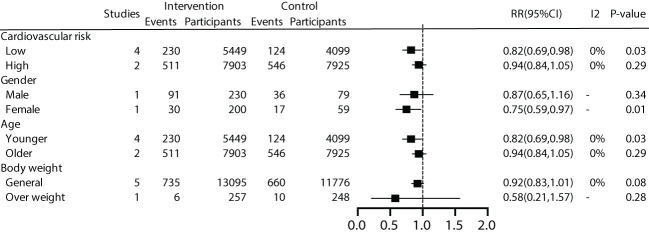
Subgroup analysis of all-cause mortality.

We further conducted subgroup analyses on patient characteristics, including a history of cardiovascular disease, gender, age, and BMI. The results revealed that early effective intervention significantly reduced all-cause mortality by 18% in prediabetic patients without a history of cardiovascular disease (RR=0.82, 95% CI=0.69~0.98, *P*=0.03, I^2^ = 0%), but not in patients with a history of cardiovascular disease (*P*=0.29). Moreover, women (*P*=0.01) and younger individuals (*P*=0.01) exhibited lower all-cause mortality rates. Subgroup analysis based on BMI showed no significant benefit in all-cause mortality for general patients (*P*=0.08) or overweight patients (*P*=0.28) (**Figure 4**).

For patients without a history of cardiovascular disease, we conducted a more in-depth analysis based on a 10-year cardiovascular risk. The results revealed that early intervention effectively reduced all-cause mortality by 21% for patients with a 10-year cardiovascular risk >10% (RR=0.79, 95% CI=0.66~0.95, *P*=0.01, I^2^ = 0%), while the effect was not significant for studies with a 10-year cardiovascular risk <10% (*P*=0.67). (**Supplementary Appendix**).

### Secondary outcomes

The results indicated that early effective intervention did not significantly reduce composite cardiovascular events (RR=0.95, 95% CI=0.84~1.08, *P*=0.42, I^2 ^= 56%), core cardiovascular events (RR=0.99, 95% CI=0.90~1.10, *P*=0.9, I^2^ = 11%), cardiovascular death (RR=0.93, 95% CI=0.81~1.06, *P*=0.28, I^2^ = 20%), occurrence of myocardial infarction events (RR=0.99, 95%, CI=0.67~1.46, *P*=0.96, I^2^ = 61%), stroke events (RR=0.93, 95% CI=0.78~1.11, *P*=0.42, I^2^ = 58%), congestive heart failure (RR=1.53, 95% CI=0.05~48.24, *P*=0.81, I^2^ = 77%), and revascularization (RR=0.96, 95% CI=0.70~1.30, *P*=0.78, I^2^ = 43%) (**Figure 3**).

**Figure 5:**
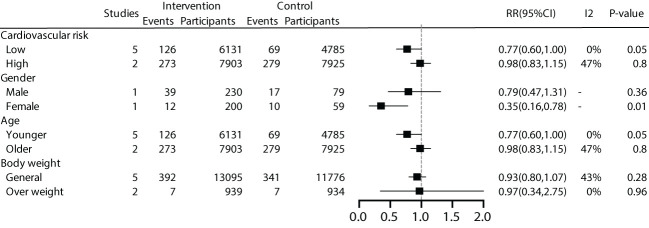
Subgroup analysis of cardiovascular mortality.

We conducted subgroup analyses based on patient characteristics, including a history of cardiovascular disease, gender, age, and BMI. The results showed that early effective intervention could significantly reduce cardiovascular mortality by 23% in patients without a history of cardiovascular disease (RR 0.77, 95% CI 0.60-1.00), although it was not statistically significant (*P*=0.05). Women exhibited lower cardiovascular mortality rates. A 23% reduction in cardiovascular mortality was also observed in younger individuals (RR 0.77, 95% CI 0.60-1.00), but it lacked statistical significance (*P*=0.05). No significant benefits were demonstrated for overweight and general populations (**Figure 5**).

For patients without a history of cardiovascular disease, we conducted a deeper analysis based on a 10-year cardiovascular risk. The results showed that effective intervention could reduce cardiovascular mortality by 26% for patients with a 10-year cardiovascular risk >10% (RR=0.74, 95% CI=0.57~0.98, *P*=0.03, I^2^ = 0%), while the effect was not significant for patients with a 10-year cardiovascular risk <10% (*P*=0.80) (**Supplementary Appendix**).

Regarding microvascular complications, the number of included studies was limited. For microvascular events, subgroup analysis was not conducted due to the limited number of studies. The overall results showed that early effective intervention could reduce retinopathy by 38% (RR=0.62, 95% CI=0.44~0.86, *P*=0.005, I^2^ = 0%), although there was a trend towards reducing kidney disease by 15%, it was not significant (*P* =0.05). Peripheral neuropathy did not demonstrate significant benefits (*P* =0.08) (**Figure 3**).

**Figure 6:**
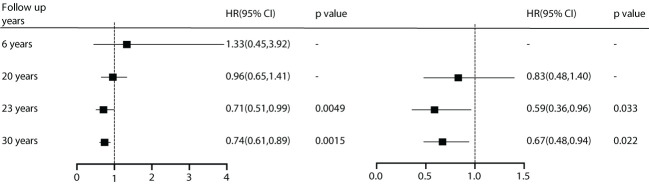
Subgroup analysis of follow-up time.

During the analysis of the included studies, we noted that the DQ study conducted data summarization and analysis at 6, 20, 23, and 30 years. Although diabetes incidence was reduced with effective intervention at all time points, microvascular events showed benefits early on. However, for cardiovascular events, there were no advantages observed in the first 20 years. Starting at 23 years, the benefits in all-cause mortality (HR) and cardiovascular mortality became significantly prominent. This suggests that besides patient characteristics, follow-up duration is also a crucial factor for effective intervention (**Figure 6**).

## Discussion

To the best of our knowledge, this is the first systematic review and meta-analysis to assess the benefits of effective intervention on microvascular and macrovascular events in prediabetic patients. Through standardized research methods, we found that, for patients without a history of cardiovascular disease, women, and younger individuals, there were lower all-cause mortality rates. In patients without a history of cardiovascular disease, for those with a 10-year cardiovascular risk >10%, there were significant reductions in all-cause mortality and cardiovascular mortality. Additionally, through longitudinal comparisons in the DQ study, we found that follow-up duration may be a critical factor in assessing effectiveness. In light of these findings, we provide evidence to guide clinical practitioners in long-term interventions for prediabetic patients.

Combining prior relevant research with our study results, it is evident that prediabetic patients require attention and early management. Some now consider prediabetes as a suboptimal health condition, and prediabetes and diabetes are seen as a continuum of elevated blood glucose and cardiovascular risk. This suggests that the principles applicable to the treatment of type 2 diabetes should also be applied to prediabetes (43, 44). Firstly, prediabetes shares the same vascular risk factors as diabetes, such as glucose abnormalities, hypertension, lipid abnormalities, obesity, and insulin resistance (44). These factors may also accelerate the development of atherosclerosis (45–47), and for prediabetic patients with comorbidities such as atrial fibrillation, there is also an increased likelihood of major cardiovascular and cerebrovascular events (48). Additionally, prediabetic patients may exhibit physiological and pathological abnormalities similar to insulin resistance (44, 49), which appears to be a significant factor in the development of cardiovascular diseases (50). For diabetic patients, the risk of cardiovascular events is 2-4 times higher than that of non-diabetic individuals (51, 52), and these risks are nearly equivalent to those of prediabetic patients (53–55).

Considering the current evidence on prediabetes management, lifestyle intervention forms the foundation. These studies collectively suggest that regardless of medication intervention, lifestyle intervention should be maintained in the long term for individuals with prediabetes. The ‘Expert Consensus on the Prevention of Type 2 Diabetes in Chinese Adults’ states that when intensive lifestyle intervention persists for more than 6 months and blood glucose control remains unsatisfactory, medication treatment may be considered for young individuals with good economic conditions, higher health needs, and access to medical services. The consensus identifies three classes of medications for prediabetes intervention: metformin, acarbose, and thiazolidinediones (TZDs). However, it emphasizes that lifestyle intervention primarily aids in weight loss, and obesity is a strong risk factor for cardiovascular diseases (56, 57), of course, this also includes type 2 diabetes (58). This is because weight loss can reduce or even reverse ectopic deposition, improve chronic inflammation, cardiovascular risk factors, and achieve the delay of disease progression (59). A 4-year follow-up observational longitudinal study indicated that weight loss surgery can significantly increase the remission rate of prediabetes (60).

The progression of prediabetes is reversible, and effective intervention is crucial for the prognosis of individuals with prediabetes (61). Combining diet and physical exercise is more effective in reducing diabetes development than any single strategy, as calorie intake and physical activity are independently associated with reducing diabetes risk, and their combination may produce an additive effect (62–64). The DQ study indicated that the reduction in diabetes incidence in the intervention group was mainly due to changes in dietary structure, increased physical activity, or improved physical fitness (13). Recent cost-effectiveness analyses have shown that lifestyle intervention is the most cost-effective approach (65). The DPP study demonstrated that intensive lifestyle intervention could reduce cardiovascular risk factors, including high blood pressure, high triglyceride levels, and low high-density lipoprotein levels (66). The effectiveness of lifestyle intervention diminishes over time, indicating the need for long-term adherence to realize significant benefits (62) However, the DQ study showed that the advantages of lifestyle intervention in reducing diabetes incidence persisted for 30 years after intensive lifestyle intervention. The DQ study provides three potential explanations for the long-term benefits of lifestyle intervention. Firstly, lifestyle intervention may lead to sustained changes in normal behavior beyond the study period. Secondly, these interventions may lead to changes in preventive care and health promotion efforts provided by community clinics, with effects observed beyond the study period. Finally, lifestyle intervention may result in a type of metabolic memory, where motivation for long-term lifestyle changes is crucial for improving intervention compliance (67). In this regard, Penn et al. suggested that an expectation of one year or longer is a necessary condition for establishing long-term behavioral changes (68). For high-risk populations with prediabetes, especially those with additional cardiovascular risk factors, medication treatment is recommended (6). Medication intervention can more rapidly reduce and stabilize risk factors in prediabetic patients. The DPP study showed that metformin did not have a treatment advantage over lifestyle intervention (29), but metformin’s effectiveness in reducing the conversion rate of prediabetes still exists (69). Changes in lifestyle may be the most effective, and the addition of pharmacological drugs does not necessarily increase the benefits. However, the STOP study also showed that early intervention with acarbose could significantly reduce cardiovascular outcomes. This could be related to the multiple benefits, including weight reduction, decreased BMI, waist circumference, blood pressure, postprandial 2-hour blood glucose, and triglyceride levels associated with acarbose. Similarly, the ACE study, which also used acarbose, did not demonstrate significant benefits, possibly due to the lower acarbose dosage and the study population’s high prevalence of coronary heart disease (22). Population-specific factors and gastrointestinal side effects should also be considered in practical application. Research indicates that acarbose appears to be more effective in preventing and reversing prediabetes in Eastern populations compared to Western populations (70). Of course, there are currently numerous clinical studies underway, including the CINEMA study which focuses on comprehensive, patient-centered, team-based interventions (71). These efforts contribute to further enriching comprehensive management strategies for prediabetes.

Our study demonstrates that long-term benefits of early effective intervention are more significant for patients without a history of coronary heart disease. This could be attributed to the fact that patients with a history of cardiovascular disease receive more combined treatments, which may not allow the advantages of intervention to be well highlighted. In the ACE study, for instance, 93% of the patients took statins, 98% took antiplatelet drugs, and 66% used beta-blockers. In the NAVIGATOR study, 39.39% of the patients used beta-blockers (which increased to 41.26% by the end of follow-up), 73.24% used antihypertensive drugs (76.35% by the end of follow-up), 38.44% used lipid-lowering medications (50.06% by the end of follow-up), and 36.80% used antiplatelet drugs (45.49% by the end of follow-up). This is significantly higher compared to studies involving individuals without a history of cardiovascular disease, such as the DREAM study, where 12.94% of the patients took statins, 14.31% took antiplatelet drugs, and 17.31% used beta-blockers. Another possible reason could be the insufficient dosage of intervention medications. The ACE study may reflect lower acarbose dosage (50 mg vs. 100 mg three times a day) compared to the STOP-NIDDM study. In the NAVIGATOR study, the most convincing evidence for improved cardiovascular outcomes with higher dosage was seen with valsartan, where patients received twice the daily dose.

Apart from lifestyle modifications, medications, and surgical interventions, it is crucial for clinical physicians to develop more rational, precise, and effective comprehensive management strategies for prediabetic patients, especially if high-risk populations, including those at risk for diabetes and cardiovascular diseases, can be accurately identified. Of course, we can refer to currently recognized relevant risk factors, including age, body mass index, family history of diabetes, history of hypertension, and physical activity level. However, more precise prediction models or scoring systems can better quantify the risks associated with prediabetic patients. For example, a recent study in China, based on data from 184,188 prediabetic patients, developed and validated personalized prediabetes prediction charts for Chinese adults, aiding in the identification of high-risk populations (72). Nevertheless, overall, there is currently no widely recommended optimal model available. Therefore, future research should focus on improving the clinical relevance and predictive performance of existing models (73, 74).

In our study, due to data limitations, there was relatively less data available for gender, age, and weight. Gender data were only available from the DQ study, and although age was categorized with a cutoff at 50 years, the NAVIGATOR and ACE studies included patients aged 50 and above. However, for the STOP study, DPP, FDPS, and DREAM, as well as NAVIGATOR and ACE, there were significant age differences. Nevertheless, we did not observe additional benefits in older individuals. It is possible that both studies included patients with a history of cardiovascular disease. Regarding gender, subgroup analysis was conducted only in the DQ study, revealing that although a reduction in mortality was primarily seen in female patients, early effective intervention did not significantly benefit males. This could be attributed to a higher number of male smokers, which may have attenuated the benefits of early intervention. Subgroup analysis based on weight revealed that for overweight patients, cardiovascular benefits were not significant. Further analysis based on an average BMI greater than 30 showed that even after excluding the DQ and ACE studies, all-cause mortality remained non-significant. Follow-up time is a key factor to consider for assessing long-term benefits. The DQ study indicated that differences in cardiovascular disease mortality between the intervention and control groups began to appear 12 years after the start of the study and slowly increased to 17% by the 20-year follow-up, reaching statistical significance only after 23 years (15). The WCK study also suggested that at the end of 10 years of treatment, there was no significant impact on mortality (75), but significant differences started to emerge when the follow-up period exceeded 22 years (76). Although the FDPS study did not show a reduced risk of cardiovascular disease over the 10-year follow-up, the incidence of type 2 diabetes in the intervention group remained significantly lower than the control group (40). This was due to a lower complication rate in the intervention group, primarily occurring over a longer time after randomization, possibly indicating a delayed onset of diabetes. This also suggests that follow-up time is a crucial factor for assessing the long-term benefits of intervention.

Our study, based on the synthesis of current high-quality research, indicates that effective intervention, especially lifestyle intervention, significantly benefits individuals with prediabetes, particularly those without a history of coronary heart disease. Subgroup analysis of the data suggests that individuals at high cardiovascular risk for 10 years, as well as women, exhibit more pronounced benefits. At present, the evidence does not clearly define age or BMI as factors influencing the effectiveness of long-term intervention. Lastly, considering the DQ study, follow-up time appears to be a crucial factor for evaluating long-term benefits of intervention. Therefore, patients should adhere to interventions in the long term, and lifestyle intervention is the most cost-effective and easily accepted approach. Given the current data limitations, detailed stratification of patients based on baseline levels such as age, weight, family history, blood pressure, and lipid profiles, which are risk factors for both macrovascular and microvascular complications, cannot be performed. Follow-up time is a critical factor, but the studies included in the analysis had varying follow-up durations, ranging from 3 to 30 years. Consequently, achieving balanced data among the groups based on current evidence may pose some challenges and potential biases. Therefore, we hope that further research will continue to address the disparity in follow-up times to obtain more reliable evidence.

It is estimated that by 2025, the global population defined as having IFG and/or IGT as prediabetes will reach 472 million (77). Individuals with prediabetes should be aware of their increased risk of future vascular complications (78). However, the vast majority of prediabetic individuals may not even be aware of their risk of developing diabetes (79). Based on the current high-quality evidence, our results indicate that early effective intervention can be used as an effective primary prevention strategy for diabetes, reducing all-cause mortality. This benefit is particularly notable in individuals without a history of cardiovascular disease, those at high cardiovascular risk for 10 years, and women. This provides a basis for clinical guidance in the intervention of individuals with prediabetes.

## Data availability statement

The original contributions presented in the study are included in the article/**Supplementary Material**. Further inquiries can be directed to the corresponding authors.

## Author contributions

XA: Data curation, Formal analysis, Writing – original draft. YZ: Data curation, Formal analysis, Writing – review & editing. WS: Methodology, Writing – review & editing. XK: Data curation, Methodology, Writing – review & editing. HJ: Investigation, Methodology, Writing – review & editing. YS: Data curation, Methodology, Writing – review & editing. LJ: Data curation, Writing – review & editing. XZ: Data curation, Writing – review & editing. QG: Data curation, Writing – review & editing. FL: Conceptualization, Writing – review & editing. XT: Conceptualization, Writing – review & editing.
