# Supplementary material for: Early effective intervention can significantly reduce all-cause mortality in prediabetic patients: a systematic review and meta-analysis based on high-quality clinical studies
Source: Front Endocrinol (Lausanne). 2024 Mar 1;15:1294819. doi: 10.3389/fendo.2024.1294819 (PMC10941028; doi:10.3389/fendo.2024.1294819)
Supplement: Supplementary file 1 [file DataSheet_1.docx]

**Supplementary appendix**

Contents

[1. Search strategy 2](#_Toc40474355)

[2. Statistical Analysis 4](#_Toc40474356)

[3. Definition of outcomes 5](#_Toc40474357)

[4. Fig 1 Flow Diagram 6](#_Toc40474358)

[5.Table 1 Basic characteristics of the including studies 7](#_Toc40474359)

[6. Table 2 Basic characteristics of the including patients 9](#_Toc40474360)

[7. Main outcome 10](#_Toc40474361)

[7.1 Total outcomes and publication bias analysis 10](#_Toc40474362)

[7.2 Subgroup analysis of 10-year cardiovascular risk 19](#_Toc40474363)

[7.3 Subgroup analysis of interventions 22](#_Toc40474364)

[7.4 Subgroup analysis of follow-up time 27](#_Toc40474365)

[Reference 30](#_Toc40474366)

# 1. Search strategy

**Pubmed Database**

((((((((((((((("Glucose Intolerance"[Mesh]) OR Glucose Intolerance[Title/Abstract]) OR Glucose Intolerances[Title/Abstract]) OR Intolerance, Glucose[Title/Abstract]) OR Intolerances, Glucose[Title/Abstract]) OR Impaired Glucose Tolerance[Title/Abstract]) OR Glucose Tolerance, Impaired[Title/Abstract]) OR Glucose Tolerances, Impaired[Title/Abstract]) OR Impaired Glucose Tolerances[Title/Abstract]) OR Tolerance, Impaired Glucose[Title/Abstract]) OR Tolerances, Impaired Glucose[Title/Abstract] Or abnormal glucose intolerance [Title/Abstract] or impaired fasting glucose [Title/Abstract])) OR (((((("Prediabetic State"[Mesh]) OR Prediabetic State[Title/Abstract]) OR Prediabetic States[Title/Abstract]) OR State, Prediabetic[Title/Abstract]) OR States, Prediabetic[Title/Abstract]) OR Prediabetes[Title/Abstract])) OR (((((("Hyperglycemia"[Mesh]) OR Hyperglycemias[Title/Abstract]) OR Hyperglycemia, Postprandial[Title/Abstract]) OR Hyperglycemias, Postprandial[Title/Abstract]) OR Postprandial Hyperglycemias[Title/Abstract]) OR Postprandial Hyperglycemia[Title/Abstract])))

And

(randomized controlled trial [Publication Type] OR

randomized [Title/Abstract] OR

placebo [Title/Abstract])

**Embase Database**

#1 ‘Impaired Glucose Tolerance’/exp

#2 ‘Impaired Glucose Tolerance’:ab,ti OR ‘abnormal glucose intolerance’:ab,ti OR ‘impaired fasting glucose’:ab,ti OR ‘Glucose Tolerance’:ab,ti

#3 #1 OR #2

#4 #3 AND ‘randomized controlled trial’/de

**Cochrane Library**

#1 MeSH descriptor: [Glucose Intolerance] explode all trees

#2 MeSH descriptor: [Prediabetic State] explode all trees

#3 (Glucose Intolerance):ti,ab,kw OR (Glucose Intolerances):ti,ab,kw OR (Intolerance, Glucose):ti,ab,kw OR (Intolerances, Glucose):ti,ab,kw OR (Impaired Glucose Tolerance):ti,ab,kw

#4 (Glucose Tolerance, Impaired):ti,ab,kw OR (Glucose Tolerances, Impaired):ti,ab,kw OR (Impaired Glucose Tolerances):ti,ab,kw OR (Tolerance, Impaired Glucose):ti,ab,kw OR (Tolerances, Impaired Glucose):ti,ab,kw

#5 (Prediabetic State):ti,ab,kw OR (Prediabetic States):ti,ab,kw OR (State, Prediabetic):ti,ab,kw OR (States, Prediabetic):ti,ab,kw OR (Prediabetes):ti,ab,kw

#6 (Hyperglycemia):ti,ab,kw OR (Hyperglycemias):ti,ab,kw OR (Hyperglycemia, Postprandial):ti,ab,kw OR (Hyperglycemias, Postprandial):ti,ab,kw OR (Postprandial Hyperglycemia):ti,ab,kw

#7 #1 or #2 or #3 or #4 or #5 or #6

# 2. Statistical Analysis

Estimated 10-year cardiovascular risk

The composite cardiovascular outcomes (cardiovascular mortality, non-fatal myocardial infarction and non-fatal stroke, hospitalization due to heart failure, hospitalization due to unstable angina pectoris) were considered for calculating cardiovascular risk. For each study, calculate the primary outcome risk for the control group, and then divide by the average follow-up time (in years) to obtain the annualized event rate. Then multiply by 10 to get the estimated event rate for 10 years.

# 3. Definition of outcomes

Composite cardiovascular outcomes: cardiovascular mortality, non-fatal myocardial infarction and non-fatal stroke, hospitalization for heart failure, hospitalization for unstable angina.

Core cardiovascular outcomes: cardiovascular mortality, non-fatal myocardial infarct

# 4. Main outcome

## 4.1 Total outcomes and publication bias analysis

Fig 1 Composite cardiovascular outcome (A Frequentist analysis forest plots; B Funnel plot)

A

B

Fig 2 Core cardiovascular outcome (A Frequentist analysis forest plots; B Funnel plot)

A

B

Fig 3 Stroke (A Frequentist analysis forest plots; B Funnel plot)

A

B

Fig 4 Myocardial infarction (A Frequentist analysis forest plots; B Funnel plot)

A

B

Fig 5 Congestive heart failure (A Frequentist analysis forest plots; B Funnel plot)

A

B

Fig 6 Revascularization (A Frequentist analysis forest plots; B Funnel plot)

A

B

Fig 7 Cardiovascular mortality (A Frequentist analysis forest plots; B Funnel plot)

A

B

Fig 8 All-cause mortality (A Frequentist analysis forest plots; B Funnel plot)

A

B

Fig 9 Microvascular complications (A Frequentist analysis forest plots; B Funnel plot)

A

B

## 4.2 Subgroup analysis of 10-year cardiovascular risk

Fig 10 Composite cardiovascular outcome

Fig 11 Core cardiovascular outcome

Fig 12 Stroke

Fig 13 Myocardial infarction

Fig 14 Congestive heart failure

Fig 15 Revascularization

Fig 16 Cardiovascular mortality

Fig 17 All-cause mortality

## 4.3 Subgroup analysis of interventions

Fig 18 Composite cardiovascular outcome

Fig 19 Core cardiovascular outcome

Fig 20 Stroke

Fig 21 Myocardial infarction

Fig 22 Congestive heart failure

Fig 23 Revascularization

Fig 24 Cardiovascular mortality

Fig 25 All-cause mortality

## 4.4 Subgroup analysis of follow-up time

Fig 26 Composite cardiovascular outcome

Fig 27 Core cardiovascular outcome

Fig 28 Stroke

Fig 29 Myocardial infarction

Fig 30 Congestive heart failure

Fig 31 Revascularization

Fig 32 Cardiovascular mortality

Fig 33 All-cause mortality

# Reference

1. Chiasson JL, Josse RG, Gomis R, Hanefeld M, Karasik A, Laakso M. Acarbose treatment and the risk of cardiovascular disease and hypertension in patients with impaired glucose tolerance: the STOP-NIDDM trial. Jama. 2003;290(4):486-94.

2. Gong Q, Zhang P, Wang J, Ma J, An Y, Chen Y, et al. Morbidity and mortality after lifestyle intervention for people with impaired glucose tolerance: 30-year results of the Da Qing Diabetes Prevention Outcome Study. Lancet Diabetes Endocrinol. 2019;7(6):452-61.

3. Gong Q, Gregg EW, Wang J, An Y, Zhang P, Yang W, et al. Long-term effects of a randomised trial of a 6-year lifestyle intervention in impaired glucose tolerance on diabetes-related microvascular complications: the China Da Qing Diabetes Prevention Outcome Study. Diabetologia. 2011;54(2):300-7.

4. Long-term effects of lifestyle intervention or metformin on diabetes development and microvascular complications over 15-year follow-up: the Diabetes Prevention Program Outcomes Study. Lancet Diabetes Endocrinol. 2015;3(11):866-75.

5. Dagenais GR, Gerstein HC, Holman R, Budaj A, Escalante A, Hedner T, et al. Effects of ramipril and rosiglitazone on cardiovascular and renal outcomes in people with impaired glucose tolerance or impaired fasting glucose: results of the Diabetes REduction Assessment with ramipril and rosiglitazone Medication (DREAM) trial. Diabetes Care. 2008;31(5):1007-14.

6. Aro A, Kauppinen A, Kivinen N, Selander T, Kinnunen K, Tuomilehto J, et al. Life Style Intervention Improves Retinopathy Status-The Finnish Diabetes Prevention Study. Nutrients. 2019;11(7).

7. Knowler WC, Sartor G, Melander A, Schersten B. Glucose tolerance and mortality, including a substudy of tolbutamide treatment. Diabetologia. 1997;40(6):680-6.
